# Supplementary material for: RNA localization and co‐translational interactions control RAB13 GTPase function and cell migration
Source: EMBO J. 2020 Sep 18;39(21):e104958. doi: 10.15252/embj.2020104958 (PMC7604616; doi:10.15252/embj.2020104958)
Supplement: Supplementary file 1 — Appendix [file EMBJ-39-e104958-s001.pdf]

APPENDIX

RNA localization and co-translational interactions control GTPase function and cell migration

Konstadinos Moissoglu[1], Michael Stueland[1], Alexander N. Gasparski[1], Tianhong Wang[1], Lisa M. Jenkins[2], Michelle L. Hastings[3] and Stavroula Mili[1*]

[1]Laboratory of Cellular and Molecular Biology, Center for Cancer Research, National Cancer Institute, NIH, Bethesda, MD, USA
[2]Laboratory of Cell Biology, Center for Cancer Research, National Cancer Institute, NIH, Bethesda, MD, USA
[3]Center for Genetic Diseases, Chicago Medical School, Rosalind Franklin University of Science and Medicine, North Chicago, IL, USA

Contents:

Appendix Figure S1: Uptake and persistence of PMOs.
Appendix Figure S2: Rab13 PMOs do not affect *Rab13* RNA levels.
Appendix Figure S3: Effect of frameshift mutation on GFP-RAB13 distribution.
Appendix Figure S4: Detection of RABIF-RAB13 interaction through PLA.

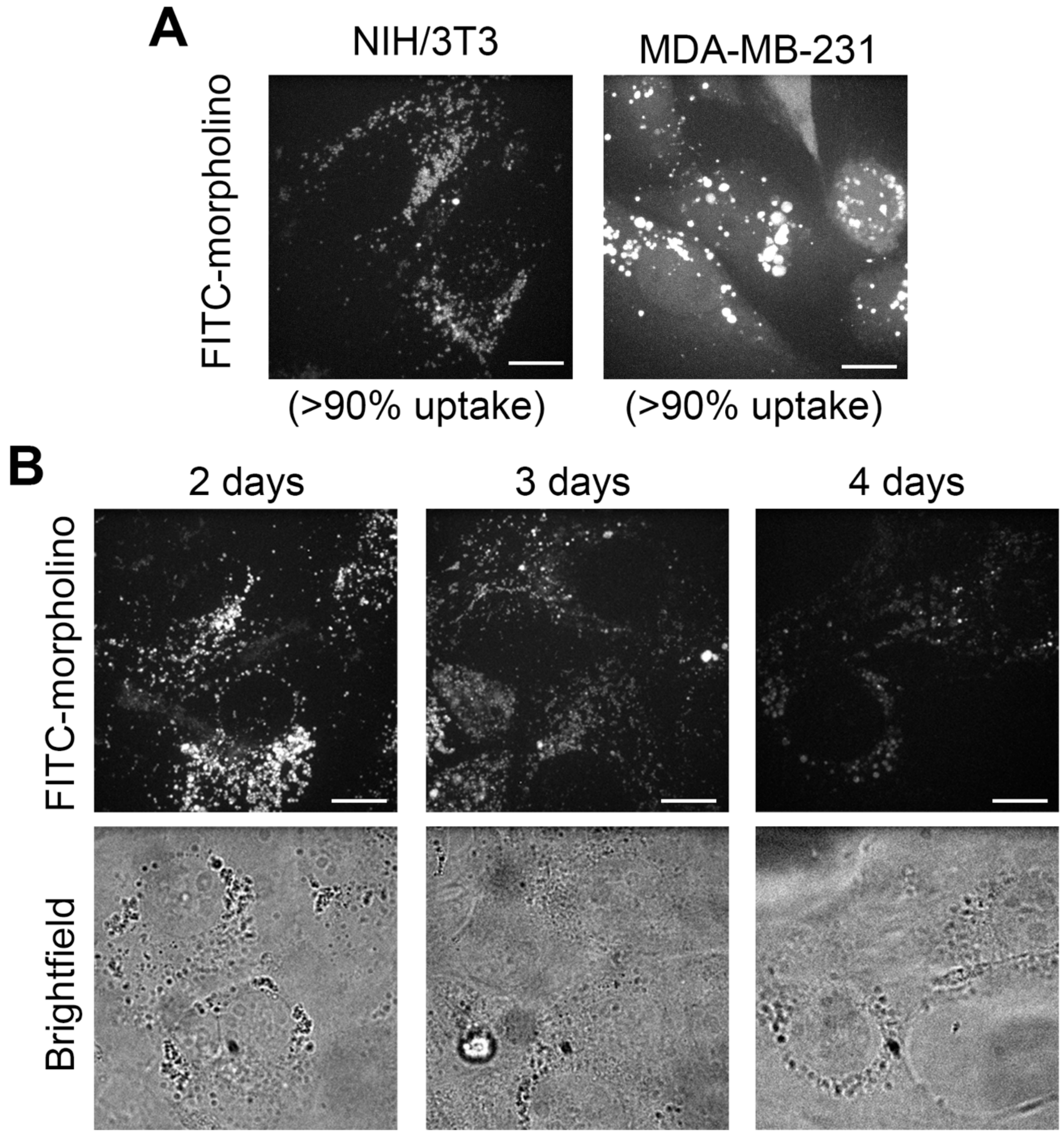

Appendix Figure S1: Uptake and persistence of PMOs.

A. FITC-labeled morpholinos were delivered into the indicated cell lines and fluorescence uptake was assessed after 24 hrs by live cell imaging. >90% of cells had visible fluorescence in intracellular vesicles or diffusely in the cytoplasm. Scale bars: 10 μm.

B. Mouse fibroblast cells were treated with FITC-labeled morpholinos. Persistence of morpholinos in cells was assessed daily for 4 days by live-cell imaging. Note that after 4 days, signal is still detectable in >90% of cells. Scale bars: 10 μm.

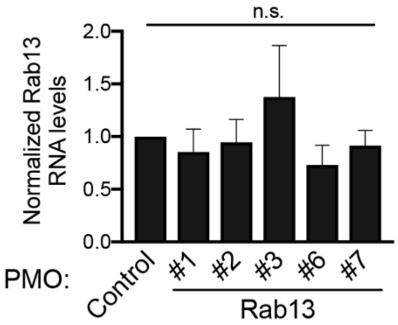

Appendix Figure S2: Rab13 PMOs do not affect *Rab13* RNA levels.

The indicated PMOs were delivered into mouse fibroblast cells. *Rab13* RNA levels were assessed by RT-ddPCR and normalized to housekeeping RNA levels. N=3-4. Bars: mean ± s.e.m.. n.s.: not significant by one-way ANOVA.

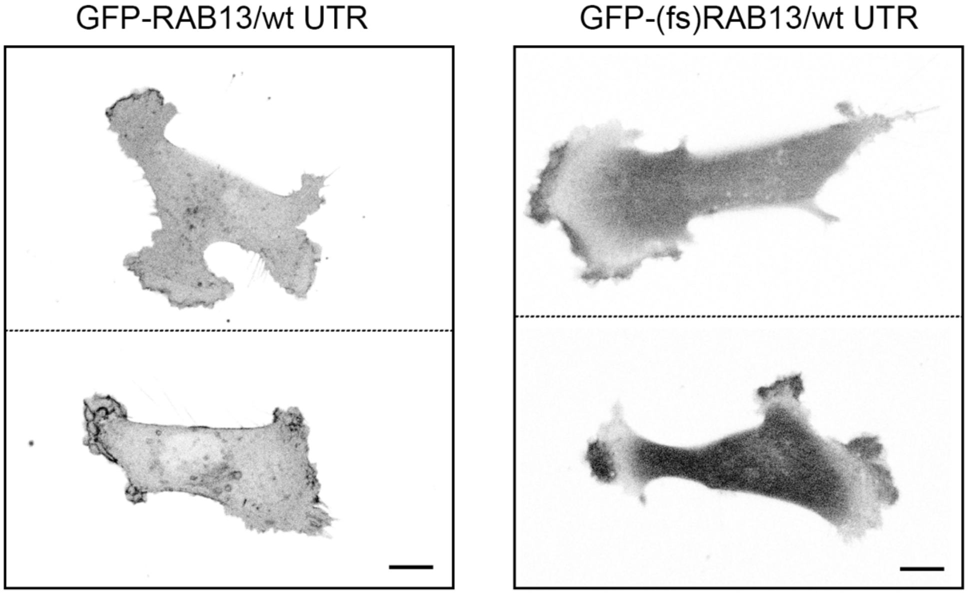

Appendix Figure S3: Effect of frameshift mutation on GFP-RAB13 distribution. Representative GFP fluorescence of GFP-RAB13/wt UTR or GFP-(fs)RAB13/wt UTR, re-expressed in RAB13 knockdown cells (see Figure 5G). The images correspond to single confocal slices at the bottom of the cell. The frameshift mutation, as expected, causes the membranous GFP-RAB13 signal to become more diffuse, cytosolic. Scale bars: 10 μm.

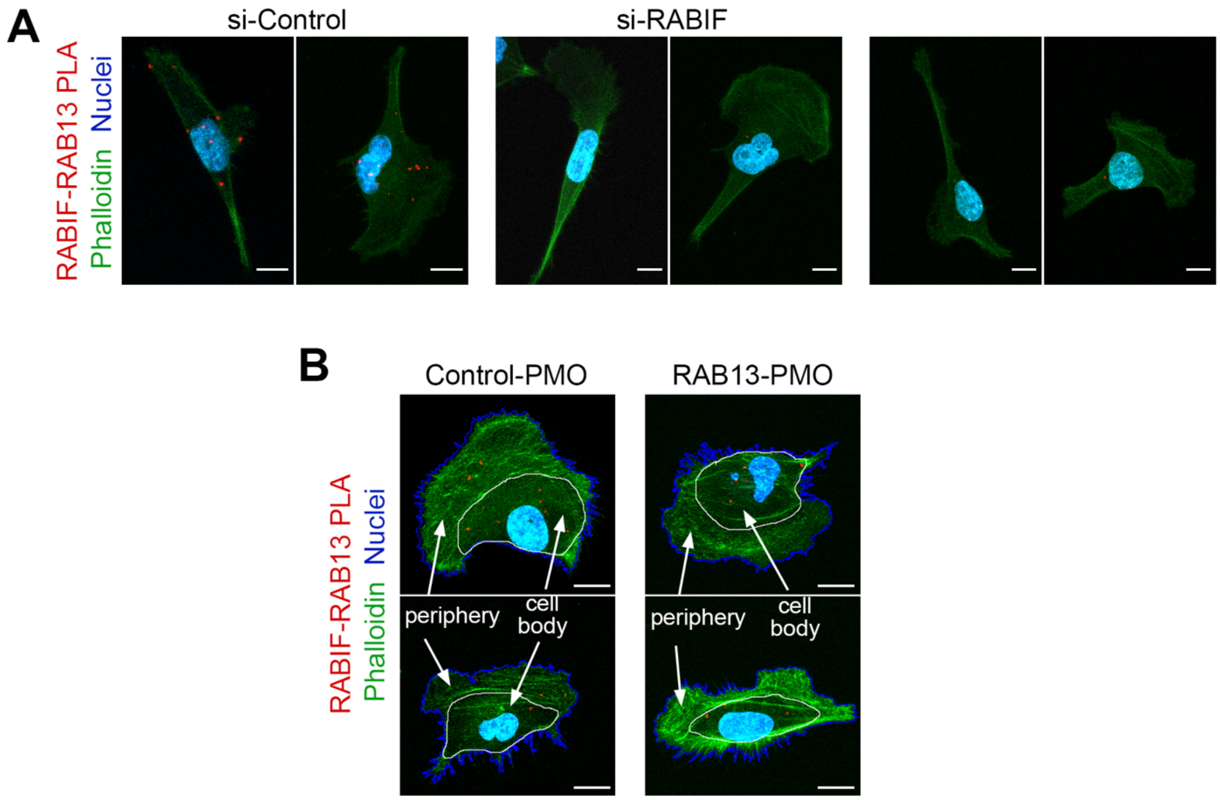

Appendix Figure S4: Detection of RABIF-RAB13 interaction through PLA.

A. Representative RABIF-RAB13 PLA images from cells transfected with the indicated siRNAs (quantified in Figure 7D). Scale bars: 10 μm.

B. Examples of sectioning of cell images into ‘peripheral’ and ‘cell body’ regions used for quantification of PLA signals in Figures 7E, F. Phalloidin staining is used to discriminate peripheral regions exhibiting denser cortical F-actin staining from perinuclear/cell body regions which exhibit less intense and more diffuse staining. Scale bars: 15 μm.
