# Supplementary material for: RNA localization and co‐translational interactions control RAB13 GTPase function and cell migration
Source: EMBO J. 2020 Sep 18;39(21):e104958. doi: 10.15252/embj.2020104958 (PMC7604616; doi:10.15252/embj.2020104958)
Supplement: Supplementary file 2 — Expanded View Figures PDF [file EMBJ-39-e104958-s002.pdf]

Expanded View Figures

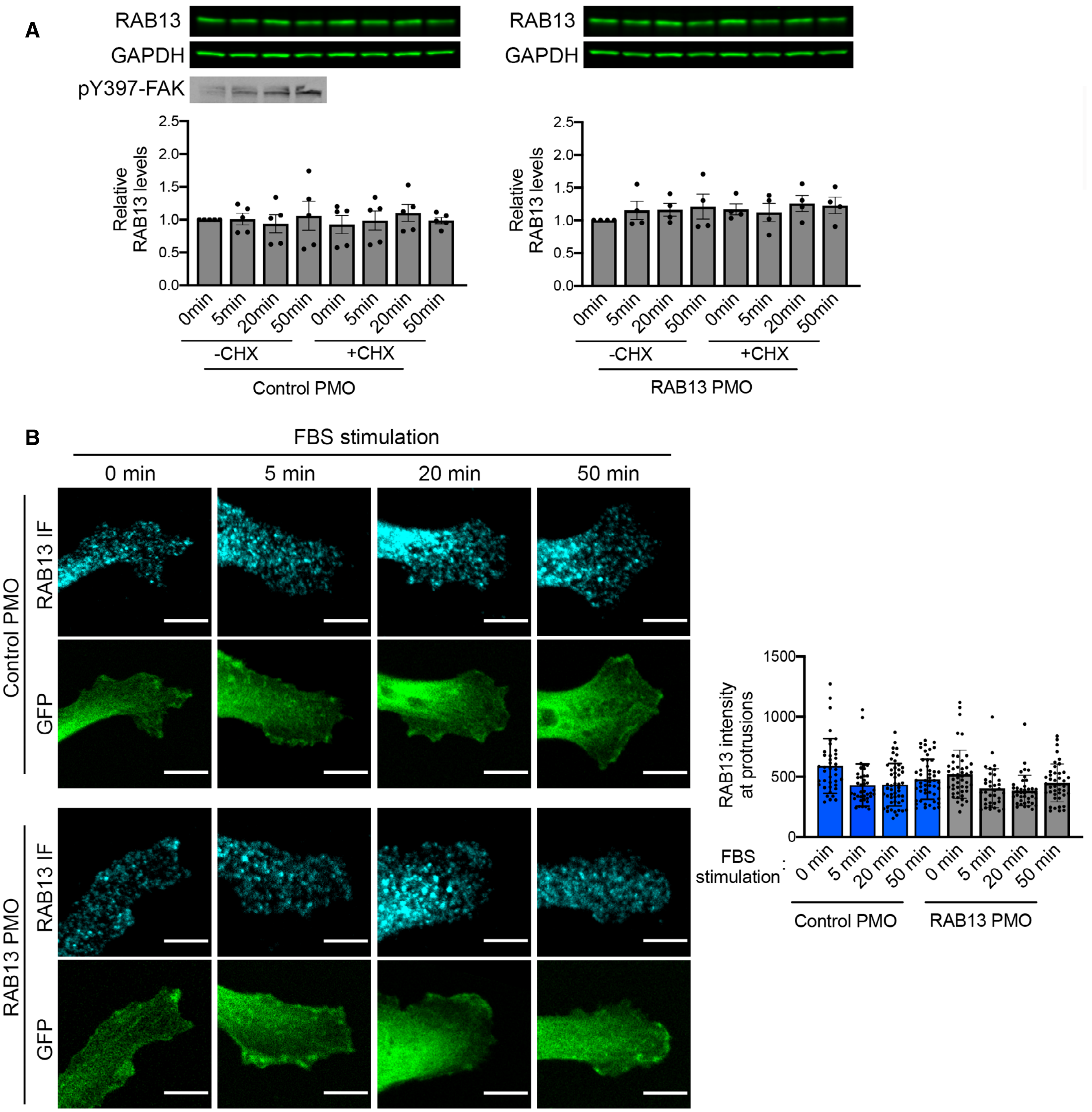

Figure EV1.

 © 2020 The Authors

◀ **Figure EV1. RAB13 protein levels do not change upon serum stimulation.**

MDA-MB-231 cells were stimulated with serum for the indicated times, in the presence or absence of cycloheximide (CHX). The cells were also treated with control or RAB13-mislocalizing PMOs.

A Representative Western blot analysis of whole-cell lysates and corresponding quantitations of RAB13 levels from n = 4–5 replicates. Bars: mean ± s.e.m. No significant differences by Friedman's test. Increase in pY397-FAK levels attests to serum stimulation.

B RAB13 immunofluorescence at representative protrusive regions. A cell line expressing GFP was used to delineate cell borders and provide an internal cytosolic control. RAB13 signal at front lamellipodial regions was quantified. n = 35–51 protrusions. Bars: mean ± s.e.m. No increase is detected upon stimulation. By contrast, at early time points a decrease is detected (5 and 20 min, $P < 0.01$ by Kruskal–Wallis test), potentially arising from serum-induced endocytosis of RAB13-containing membranes. Scale bars: 8 μm.

© 2020 The Authors

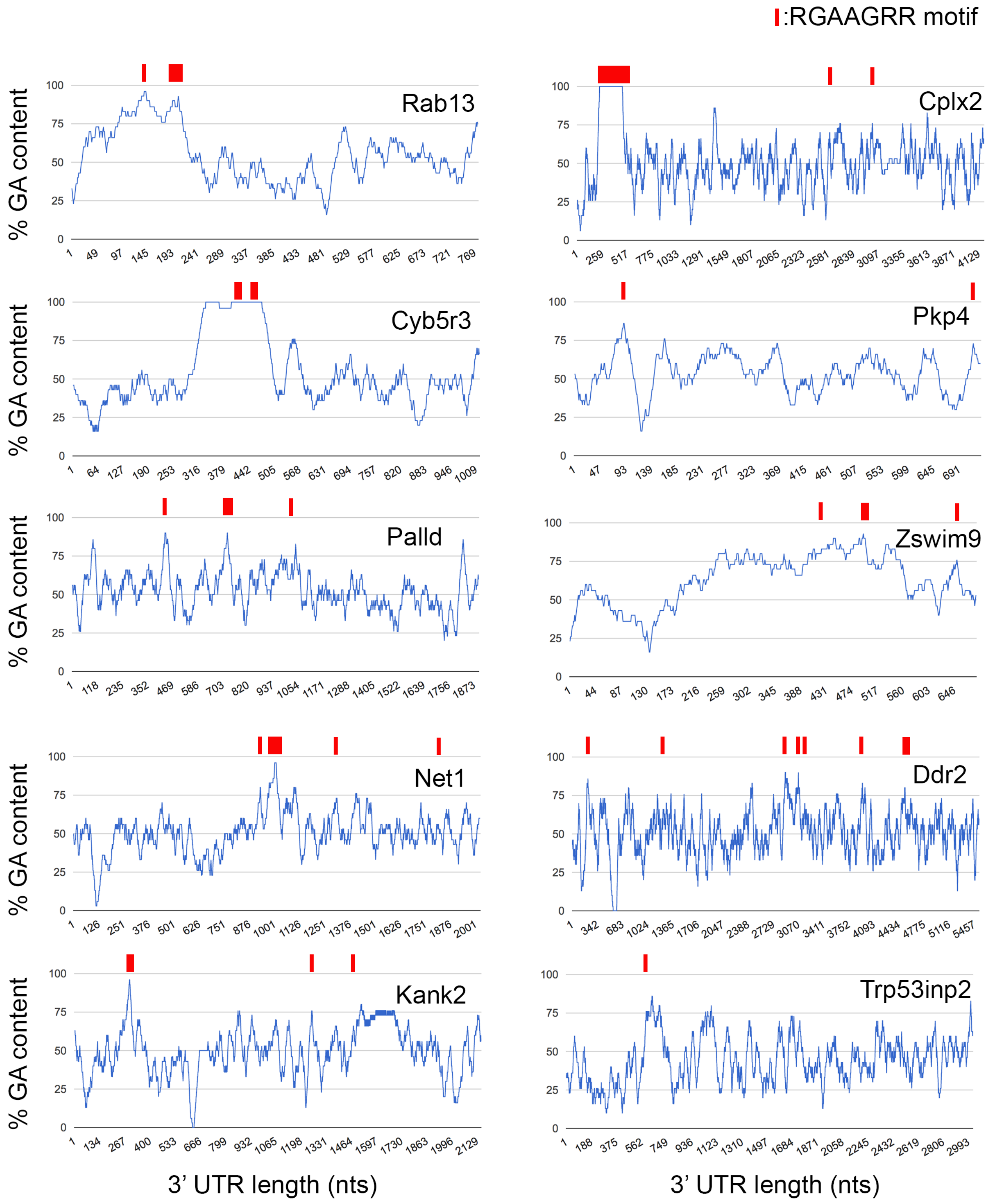

Figure EV2. GA-rich motif distribution in 3′UTRs of APC-dependent RNAs.

Graphs show the % GA content along the 3′UTR of the indicated APC-dependent RNAs using a 30-nt window size. The graph showing the Rab13 UTR is the same presented in Figs 2A and 3A. Occurrences of the consensus GA-rich motif are indicated by a red rectangle. Wider rectangles indicate the presence of multiple motifs. Exact sizes are not to scale due to the variable UTR lengths. The majority of GA-rich motifs are found within more extended GA-rich regions with GA content > 75%. Note that the GA motif is 7 nts, while the window for %GA calculation is 30 nts.

 © 2020 The Authors

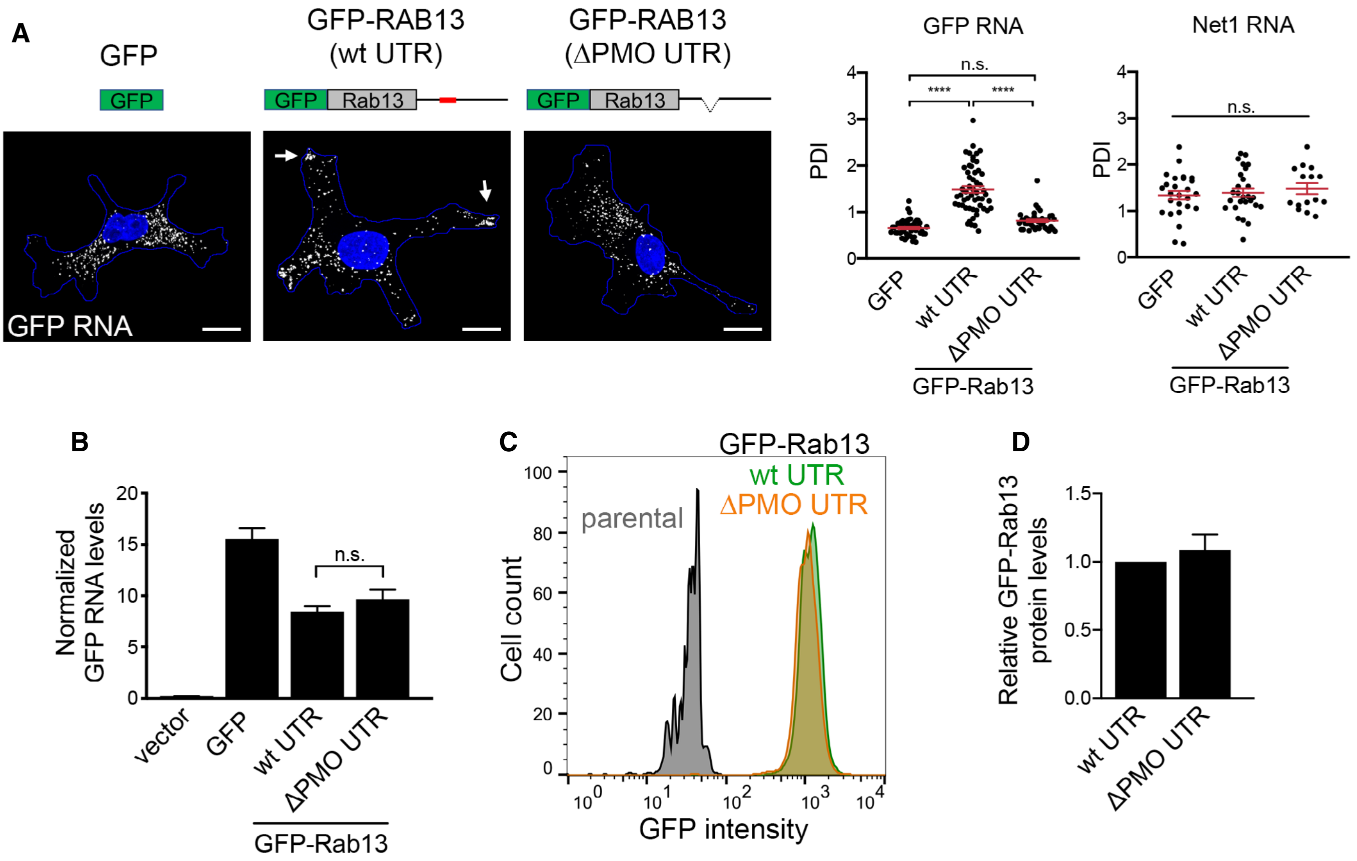

Figure EV3. Peripheral localization of exogenous *RAB13* RNA does not affect *RAB13* RNA stability or translation.

A Schematics depict GFP or GFP-RAB13 constructs stably expressed in MDA-MB-231 cells. RAB13 coding sequence is followed either by the wild-type RAB13 UTR or by the RAB13 UTR carrying a 52-nt deletion corresponding to the region targeted by PMOs 191 and 230 (ΔPMO UTR). Exogenous RNA is detected by FISH against the GFP sequence. Arrows point to RNA localized at protrusions. Scale bars: 10 μm. Graphs show PDI measurements of *GFP* or *NET1* RNA from multiple cells. n = 42–54 cells in 4 independent experiments (for GFP); n = 27 cells in 2 experiments (for Net1). Bars: mean ± s.e.m. ****$P < 0.0001$ by analysis of variance with Dunnett's multiple comparisons test.

B Levels of *GFP* RNA from the indicated cell lines were assessed by RT-ddPCR and normalized to housekeeping control RNAs. n = 6. Bars: mean ± s.e.m. n.s.: not significant by one-way ANOVA.

C The indicated cell lines were analyzed by flow cytometry to assess GFP intensity per cell.

D GFP-RAB13 protein levels of the indicated cell lines were assessed by quantitative Western blot and normalized to α-tubulin levels. n = 7. Bars: mean ± s.e.m. n.s.: not significant by Wilcoxon matched-pairs signed-rank test.

© 2020 The Authors

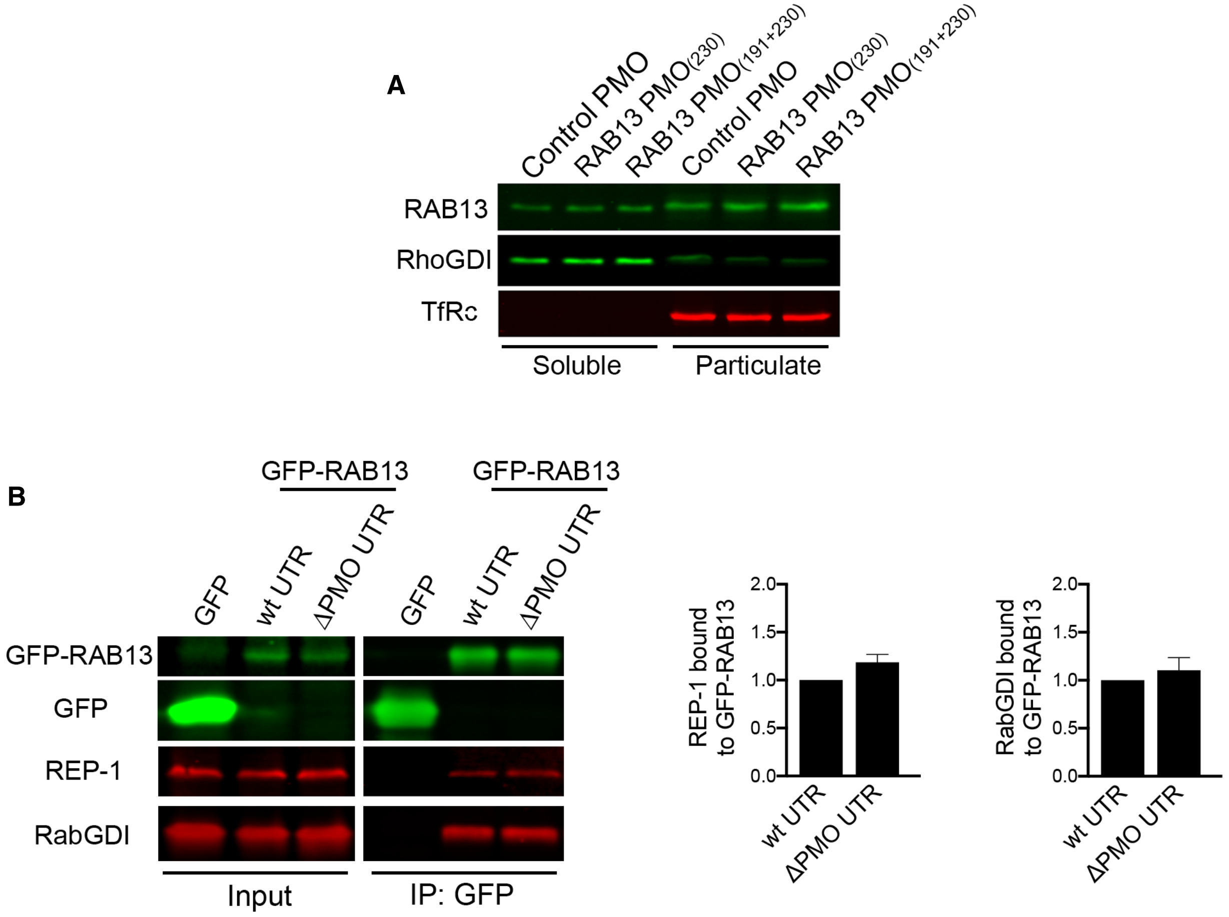

Figure EV4. *RAB13* RNA mislocalization does not affect RAB13 binding to membranes or association with REP-1 or RabGDI.

A Cells treated with the indicated PMOs were fractionated into soluble and particulate fractions, and the indicated proteins were detected by Western blot. RhoGDI and TfRc serve as soluble and particulate markers, respectively.

B Lysates from the indicated GFP or GFP-RAB13-expressing cell lines were immunoprecipitated with anti-GFP antibodies and blotted to detect the indicated proteins. Relative REP-1 and RabGDI binding are quantified in the graphs from n = 3 (REP-1) and n = 5 (RabGDI) independent experiments. Bars: mean ± s.e.m. No significant differences were detected by Wilcoxon matched-pairs signed-rank test.

© 2020 The Authors

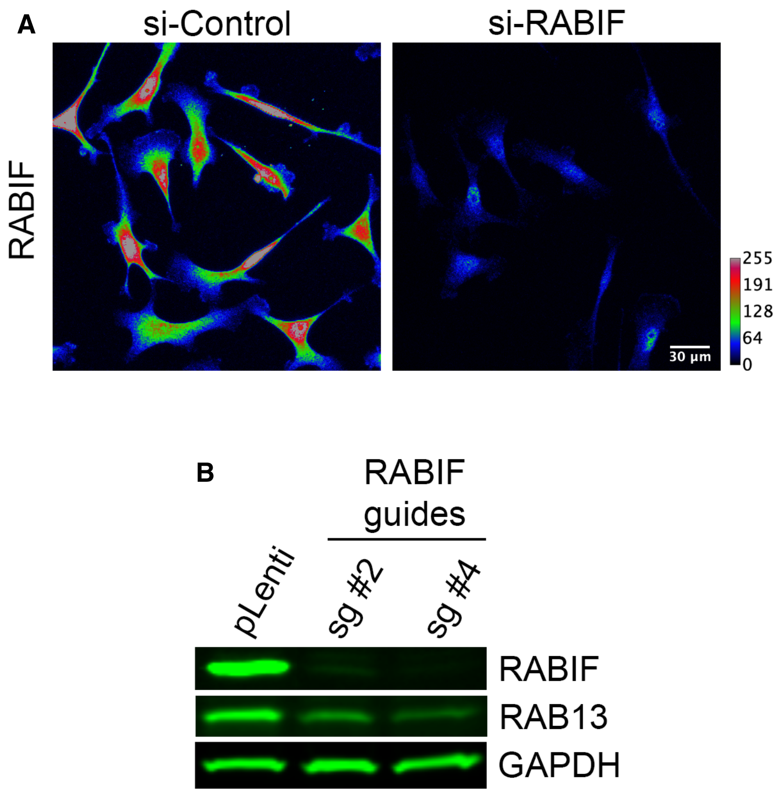

Figure EV5. RABIF distribution and effect on RAB13 expression.

A Representative immunofluorescence images of RABIF protein in cells transfected with the indicated siRNAs. Reduction of intensity in knockdown cells confirms the specificity of the signal. Calibration bar shows intensity values. Note that RABIF exhibits a mostly perinuclear enrichment.

B RAB13 expression in cells with CRISPR knockdown of RABIF using the indicated sgRNAs (see also Fig 7C).

© 2020 The Authors
