# Supplementary material for: RNA localization and co‐translational interactions control RAB13 GTPase function and cell migration
Source: EMBO J. 2020 Sep 18;39(21):e104958. doi: 10.15252/embj.2020104958 (PMC7604616; doi:10.15252/embj.2020104958)
Supplement: Supplementary file 5 — Movie EV1 [file EMBJ-39-e104958-s005.zip › Movie EV1 Legend.docx]

**Movie EV1:** Representative time lapse imaging of MDA-MB-231 cells expressing Cherry-NLS to mark nuclei. Cells plated on collagen IV-coated coverglass were imaged over time and nuclei were tracked. Tracks of individual cells are overlaid in different colors. Time stamp in Hours:Minutes.
