# Supplementary material for: RNA localization and co‐translational interactions control RAB13 GTPase function and cell migration
Source: EMBO J. 2020 Sep 18;39(21):e104958. doi: 10.15252/embj.2020104958 (PMC7604616; doi:10.15252/embj.2020104958)
Supplement: Supplementary file 6 — Movie EV2 [file EMBJ-39-e104958-s006.zip › Movie EV2 Legend.docx]

**Movie EV2:** Left panel: Time lapse imaging of a control cell, expressing Lifeact-GFP, plated on collagen IV-coated coverglass and imaged every minute over 1 hr. Right panel: Corresponding edge velocity map, with negative values indicating retraction and positive values indicating extension.
