# Supplementary figures and images for: RNA localization and co‐translational interactions control RAB13 GTPase function and cell migration
Source: EMBO J. 2020 Sep 18;39(21):e104958. doi: 10.15252/embj.2020104958 (PMC7604616; doi:10.15252/embj.2020104958)

# Source data: Figure 5E

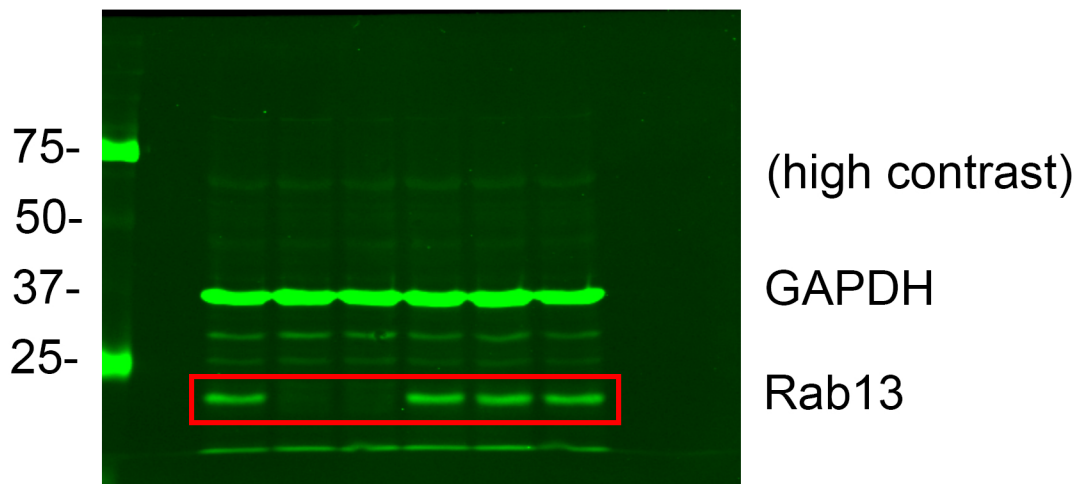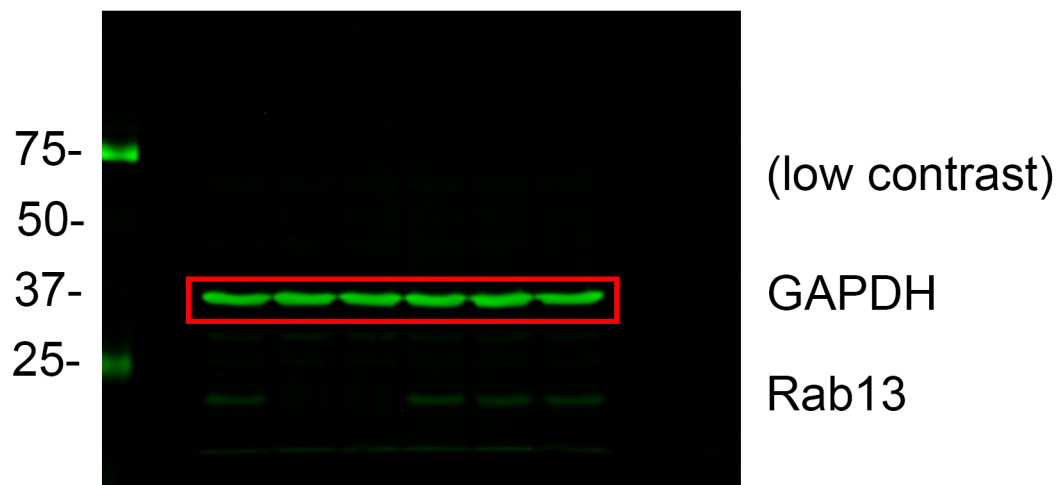

Supplement: Supplementary file 9 — Source Data for Figure 5 [file EMBJ-39-e104958-s008.pdf]

Source data: Figure 6C

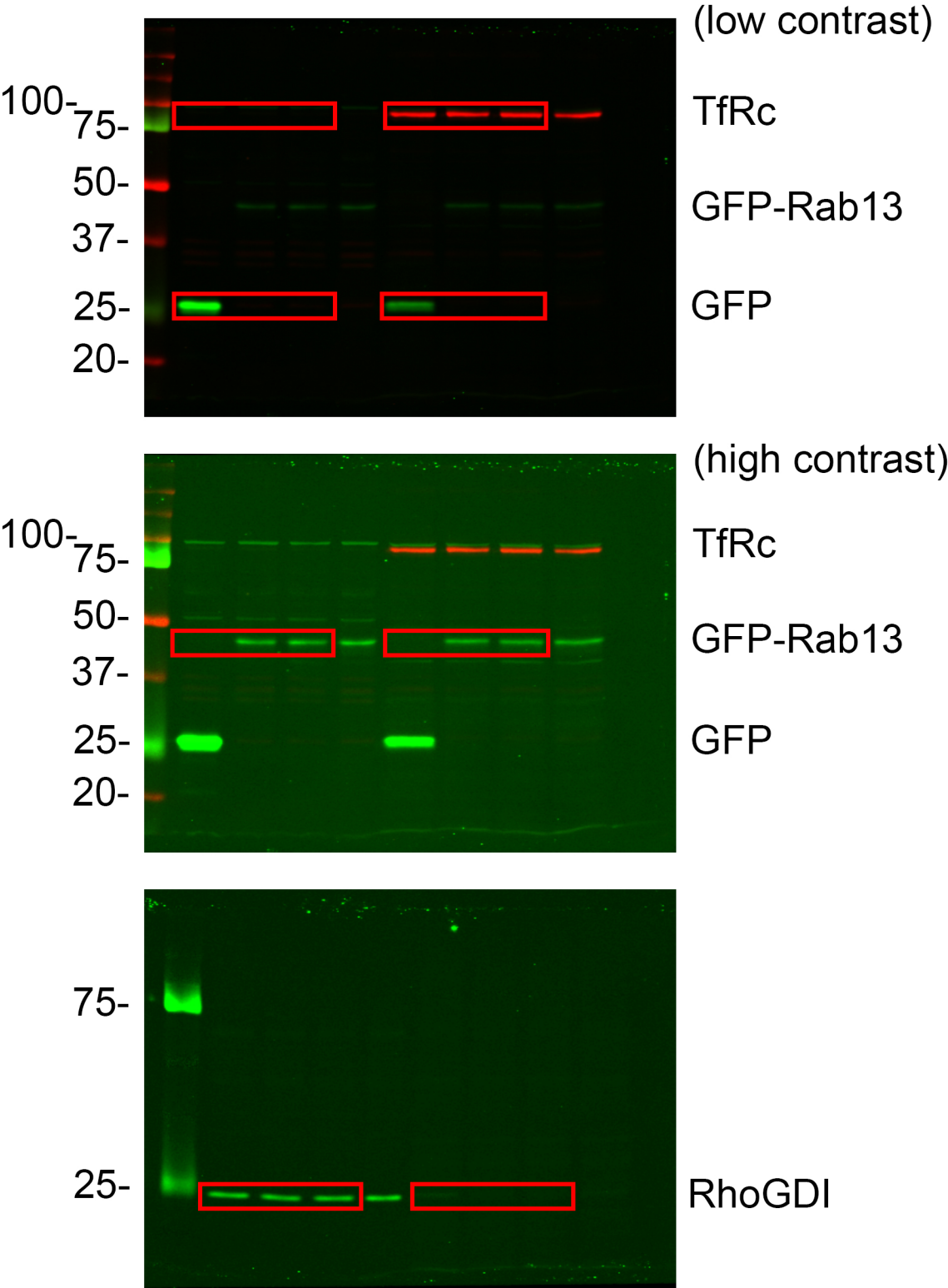

# Source data: Figure 6D

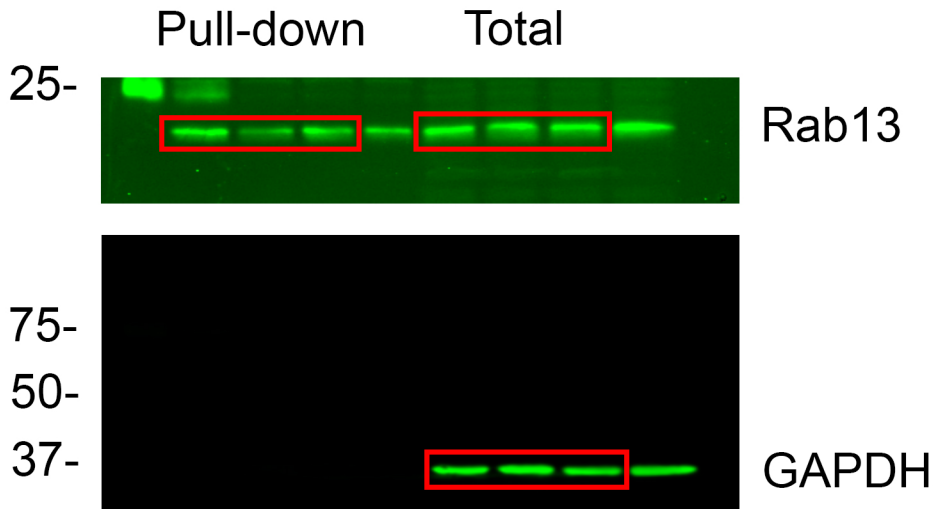

Source data: Figure 6E

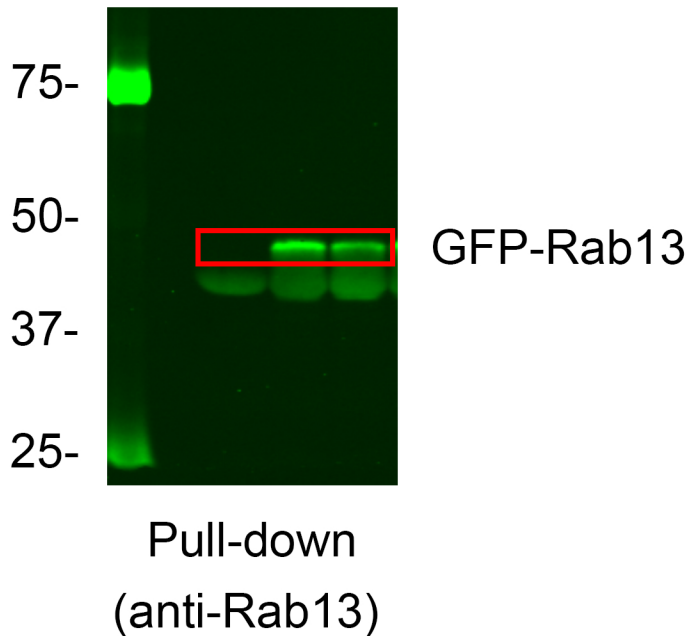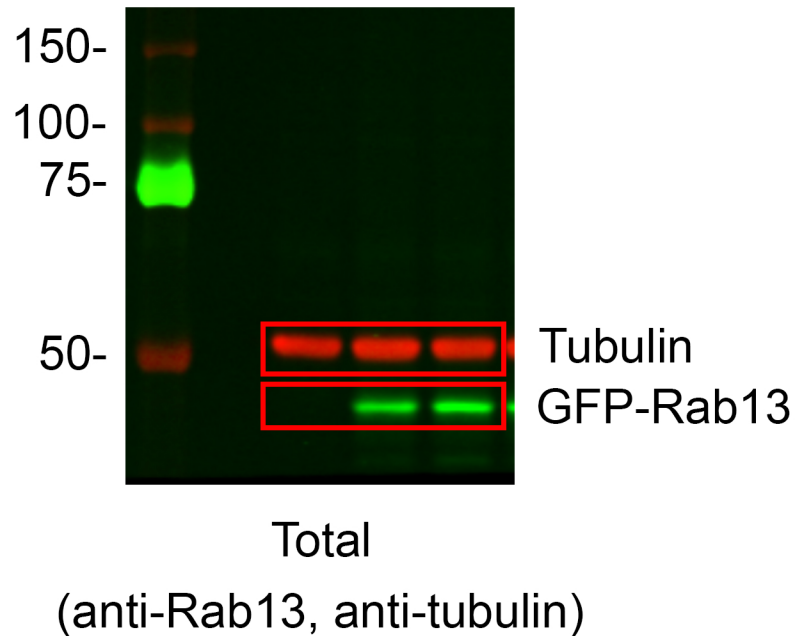

Supplement: Supplementary file 10 — Source Data for Figure 6 [file EMBJ-39-e104958-s009.pdf]

Source data: Figure 7A

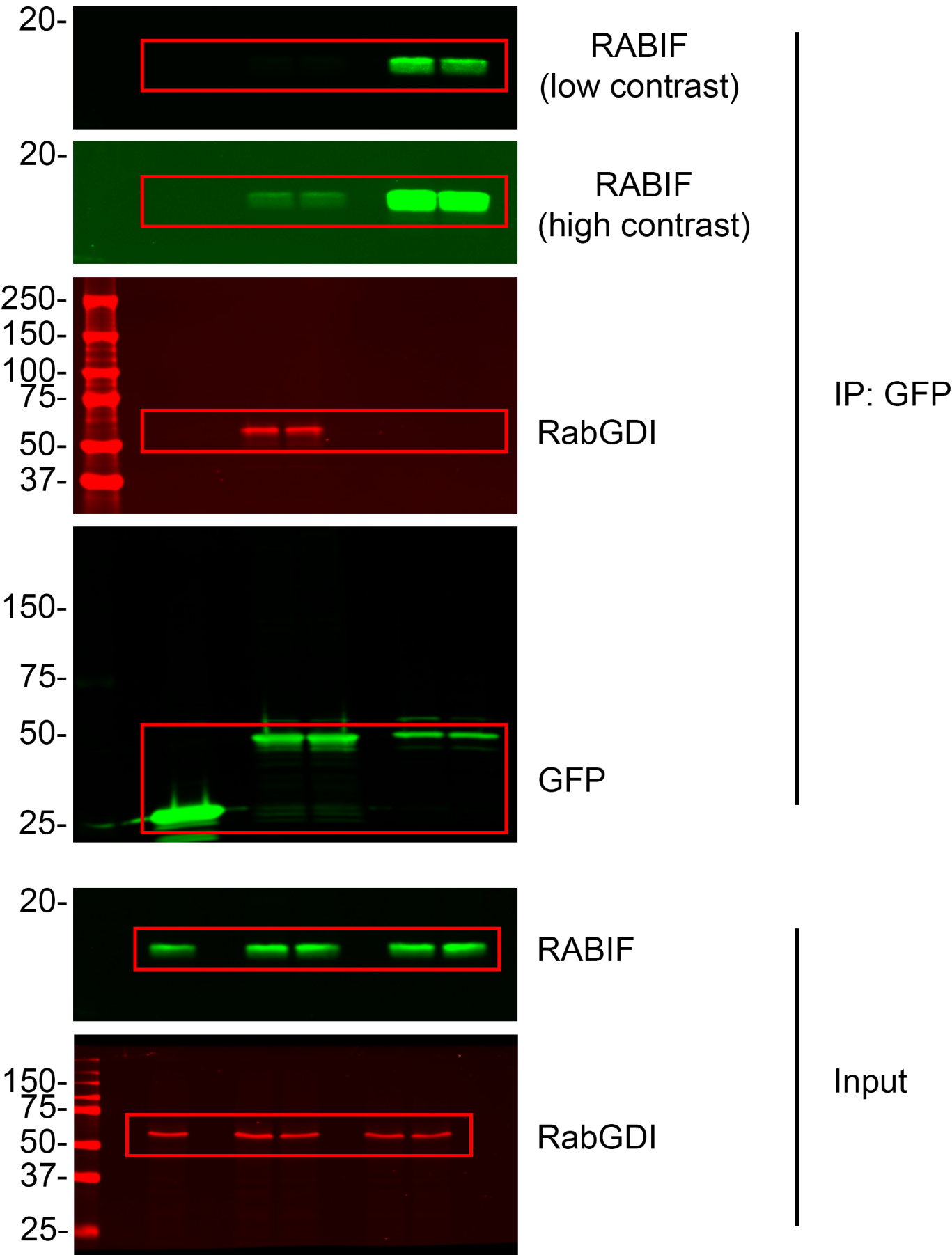

Supplement: Supplementary file 11 — Source Data for Figure 7 [file EMBJ-39-e104958-s010.pdf]
